# Optimal Distribution and Utilization of Donated Human Breast Milk: A Novel Approach

**DOI:** 10.1177/0890334416653738

**Published:** 2016-06-30

**Authors:** Judith H. Simpson, Lorna McKerracher, Andrew Cooper, Debbie Barnett, Emma Gentles, Lorraine Cairns, Konstantinos Gerasimidis

**Affiliations:** 1 Neonatal Intensive Care Unit, Royal Hospital for Sick Children, Glasgow, UK; 2 NHS Greater Glasgow & Clyde Donor Breast Milk Bank, Southern General Hospital, Glasgow, UK; 3 Neonatal Intensive Care Unit, Southern General Hospital, Glasgow, UK; 4 School of Medicine, University of Glasgow, UK

**Keywords:** breastfeeding, breast milk expression, intensive care units, milk banks, neonatal, nutrition

## Abstract

**Background:**

The nutritional content of donated expressed breast milk (DEBM) is variable. Using DEBM to provide for the energy requirements of neonates is challenging.

**Objective:**

The authors hypothesized that a system of DEBM energy content categorization and distribution would improve energy intake from DEBM.

**Methods:**

We compared infants’ actual cumulative energy intake with projected energy intake, had they been fed using our proposed system. Eighty-five milk samples were ranked by energy content. The bottom, middle, and top tertiles were classified as red, amber, and green energy content categories, respectively. Data on 378 feeding days from 20 babies who received this milk were analyzed. Total daily intake of DEBM was calculated in mL/kg/day and similarly ranked. Infants received red energy content milk, with DEBM intake in the bottom daily volume intake tertile; amber energy content milk, with intake in the middle daily volume intake tertile; and green energy content milk when intake reached the top daily volume intake tertile.

**Results:**

Actual median cumulative energy intake from DEBM was 1612 (range, 15-11 182) kcal. Using DEBM with the minimum energy content from the 3 DEBM energy content categories, median projected cumulative intake was 1670 (range 13-11 077) kcal, which was not statistically significant (*P* = .418). Statistical significance was achieved using DEBM with the median and maximum energy content from each energy content category, giving median projected cumulative intakes of 1859 kcal (*P* = .0006) and 2280 kcal (*P* = .0001), respectively.

**Conclusion:**

Cumulative energy intake from DEBM can be improved by categorizing and distributing milk according to energy content.

## Well Established

*Donated human milk is now recommended for preterm infants when maternal milk is insufficient or unavailable. Routine macronutrient analysis of human milk has been described and may serve as a means with which to improve neonatal nutrition management.*

## Newly Expressed

*It is possible to improve cumulative energy intake obtained from donated breast milk if the milk is categorized according to energy content and distributed according to milk intake volumes.*

## Background

Not only is adequate nutrition integral to growth and development in infancy, there is a growing evidence base supporting the effect of early nutrition on longer term health outcomes.^1,2^ Compared to infant formula, maternal breast milk is associated with reduced morbidity in the preterm population. This makes it the most desired choice for providing nutrition in sick and premature babies,^3,4^ but it may not be available in sufficient quantities.

Several influential health professional bodies, including the American Academy of Paediatrics^5^ and the European Society of Paediatric Gastroenterology and Nutrition,^6^ now endorse the use of donated expressed breast milk (DEBM) over infant formula milk in instances where maternal breast milk is insufficient or unavailable. However, concerns have been raised regarding the nutritional content of DEBM and, in particular, how DEBM might best meet the increased energy and nutrient requirements of sick and preterm neonates.^7,8^

Nutrition analysis of all donated milk was introduced as routine practice in the Greater Glasgow and Clyde Donor Milk Bank in 2012, and we have recently described the nutrition variability of our product.^7^ This information has provided us with the opportunity to explore how best to distribute our milk to ensure that it is received by those who need it most and who are more likely to benefit from the nutrient-dense milk. In this article, we describe a novel, user-friendly system of DEBM energy content categorization and distribution and a proof of concept evaluation of the effect of this new system on energy intake from DEBM during hospital stay.

## Methods

### Development of a Milk Categorization and Distribution System

Nutrition analysis was routinely performed on all milk samples donated to the Greater Glasgow and Clyde Donor Milk Bank using the MIRIS Human Milk Analyzer (Miris® AB) as previously described.^7^ The energy content (kcal/100 mL) and macronutrient analysis information were retrieved for 85 sequential milk samples donated to the bank between January and August 2012. The energy content of these samples was then ranked by ascending order and the bottom, middle, and top tertiles were classified as red, amber, and green energy content categories, respectively.

To establish a system of milk distribution, we collected data on 378 feeding days from 20 babies who had received milk from the 85 samples described above in an unselected manner as part of routine clinical practice. The total intake of DEBM for each day was calculated in mL/kg/day and then ranked by ascending order. This provided the definition of bottom, middle, and top daily volume intake tertiles. We proposed that babies would receive red energy content milk when their enteral intake was within the bottom daily volume intake tertile, amber energy content milk when their intake fell into the middle daily volume intake tertile, and green energy content milk when they received intakes in the top daily volume intake tertile.

### Effect of the New Traffic Light Distribution System on DEBM Energy Intake

The 20 babies who were identified from the donor milk bank database had their medical records reviewed, and characteristics such as gestation, birth weight, indication for DEBM, and concurrent use of parenteral nutrition or alternative milk were recorded. In a proof of concept evaluation, their actual or true cumulative energy intake from unselected DEBM was compared with their projected energy intakes if they had been fed according to our proposed categorization system.

Data were presented with median (interquartile range) for continuous variables and with counts and frequencies for categories. Differences between the true and projected cumulative energy intake were compared with the 1-Sample Wilcoxon Signed Rank test. Correlations between variables were explored with Pearson correlation. Data were analyzed using MINITAB 16 and the significance level was set at *P* < .05.

Prior permission to review medical records was granted by the Clinical Governance Unit, Royal Hospital for Sick Children, Glasgow. All mothers who donate milk to the Greater Glasgow and Clyde Milk Bank consent to the use of their milk in nutrition research as part of the recruitment process, and given the service evaluation nature of this work, informed consent from the parents of recipient babies was not sought.

## Results

**Figure 1.**
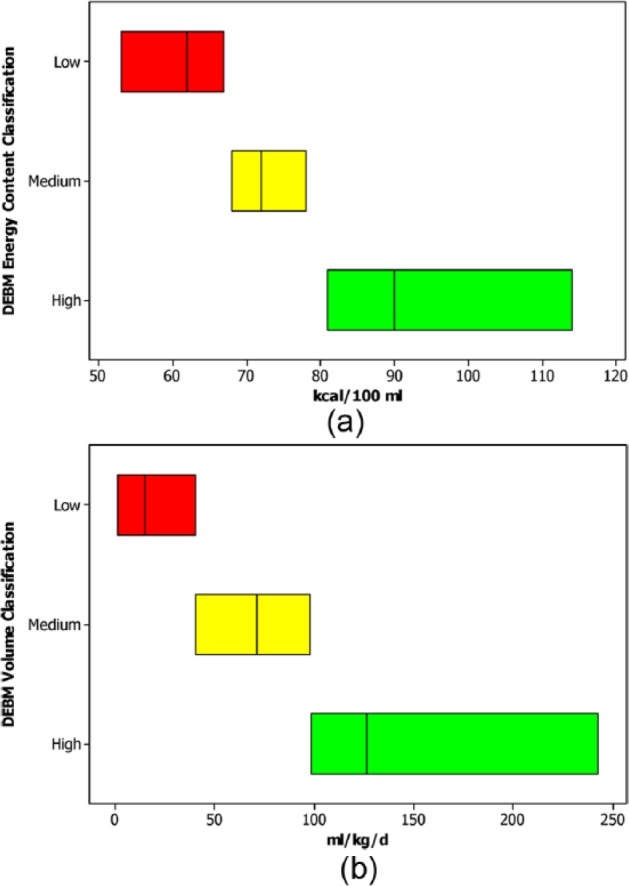
Traffic Light System for Categorization of Donated Expressed Breast Milk (DEBM) Energy Content (a) and Categorization of Total Daily Intake of DEBM (b).

The energy content of the 85 analyzed milk samples ranged from 53 to 114 kcal/100 mL, with a median of 72 kcal/100 mL. The red energy content milk contained < 68 (median = 62) kcal/100 mL, the amber energy content milk contained between 68 and 78 (median = 72) kcal/100 mL, and the green energy content milk contained > 78 (median = 90) kcal/100 mL (Figure 1a). The DEBM intake during the 378 feeding days analyzed ranged from 1.7 to 242.4 mL/kg/day, with the bottom daily volume intake tertile ranging from 1.7 to 40.7 (median = 15.0) mL/kg/day, the middle daily volume intake tertile from 40.7 to 98.1 (median = 71.2) mL/kg/day, and the top daily volume intake tertile from 98.5 to 242.4 (median = 126.4) mL/kg/day (Figure 1b).

**Figure 2.**
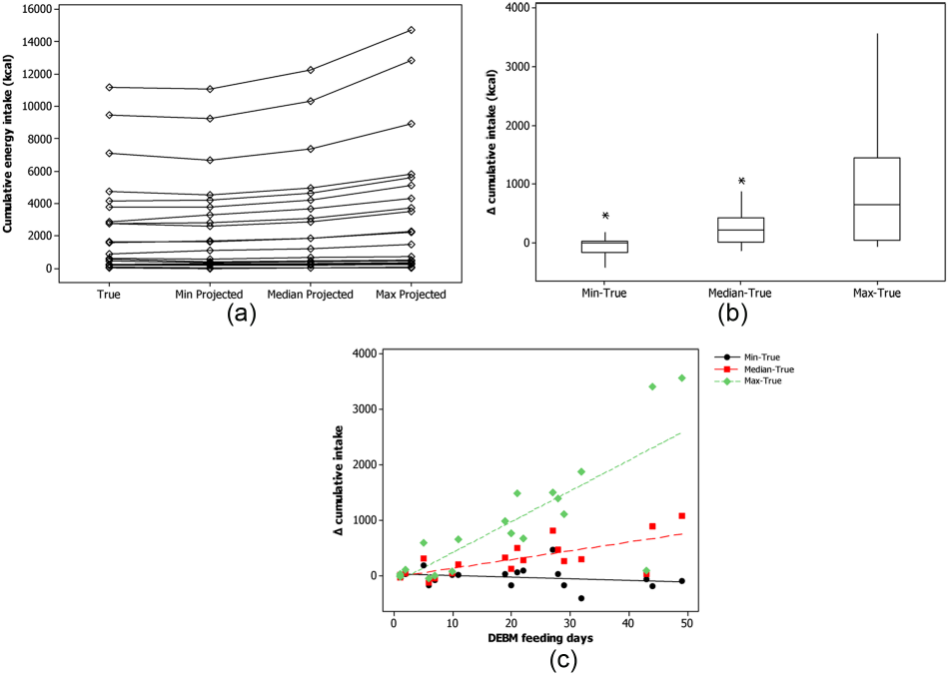
Effect of the Donated Expressed Breast Milk (DEBM) Traffic Light System on Cumulative Energy Intake. True intake versus projected intake using minimum, median, and maximum energy milk from within each DEBM energy content category. Per infant (a). Change in energy intake per minimum, median, and maximum energy content milk (b) and association with duration of DEBM feeding (c).

The characteristics of the 20 recipient babies are described in Table 1. The number of DEBM feeding days per baby varied from 1 to 29. The median true cumulative energy intake from DEBM during hospital stay was 1612 (range, 15-11 182) kcal. Using the minimum energy content from each of the DEBM categories (red, amber, and green energy content categories), the median projected cumulative intake was 1670 (range, 13-11 077) kcal, which was not significantly different from the true intake (difference true-projected, median [interquartile range (IQR)]: –1.1 [–154 to 30]; *P* = .418). Using the median energy content for milk from each energy content category, the median projected cumulative intake increased to 1859 kcal (difference true-projected, median [IQR]: 230 [16-430]; *P* = .0006) and correspondingly to 2280 kcal (difference true-projected, median [IQR]: 659 [43-1456]; *P* < .00001) when energy intake was estimated using the projected maximum energy content from each milk energy content category (Figures 2a & 2b).

**Table 1.** Characteristics of Infants Who Received DEBM.^a^

Infant	Gestation, w	Birth Weight, g	Indication for DEBM	Days on Concomitant Nutrition^b^	Days on DEBM	Days per Energy Content Category		
						Red	Amber	Green
1	24^+3^	764	Following NEC surgery	18	21	7	5	9
2	26^+4^	1050	Medical NEC	49	49	15	17	17
3	27	1045	Following NEC surgery	27	27	9	11	7
4	27^+2^	1001	Medical NEC	19	19	6	6	7
5	29^+4^	1095	Medical NEC	19	22	10	5	7
6	29^+5^	1370	Preterm	4	11	1	2	8
7	29^+6^	712	Medical NEC	23	28	11	8	9
8	30^+2^	2190	Following NEC surgery	1	1	1	0	0
9	30^+6^	1430	Following NEC surgery	10	10	9	1	0
10	31^+2^	1492	Congenital heart disease	4	6	3	1	2
11	32^+5^	1460	Following GI surgery	2	2	0	1	1
12	33	1730	Other	32	44	7	10	27
13	33^+5^	1650	Following GI surgery	16	19	3	7	9
14	34^+5^	1970	Following GI surgery	23	29	10	12	7
15	35	1500	Following GI surgery	44	44	44	0	0
16	37^+3^	2580	Following GI surgery	5	5	1	0	4
17	38^+5^	3490	Congenital heart disease	1	1	1	0	0
18	39^+2^	3370	Congenital heart disease	7	7	6	1	0
19	40	2460	Following GI surgery	23	32	5	12	15
20	41^+1^	3380	Congenital heart disease	1	1	0	1	0

Abbreviations: DEBM, donated expressed breast milk; GI, gastrointestinal; NEC, necrotizing enterocolitis.

a N = 20.

b Nutrition in addition to DEBM included parenteral nutrition, formula, or maternal expressed breast milk.

Using the new categorization system, 17 of 20 babies would have a higher cumulative energy intake from DEBM during their hospital stay if they received either medium or high energy content milk from each energy content category (Figure 2a). If they received the lowest energy content milk in each energy content category, 50% of the babies would receive more and 50% less energy (Figure 2a). The greater the number of DEBM feeding days, the more likely the babies were to have a higher projected intake compared with their true intake for the maximum (*r* = 0.73, *P* < .0001) and median energy milk (*r* = 0.79, *P* < .0001) per energy content category (Figure 2c). This was not the case for the milk with minimum energy content per energy content category (*r* = −0.23, *P* < .323) (Figure 2c).

## Discussion

With improved neonatal survival there is increased focus on longer term health outcomes and widespread recognition of the important role that early nutrition plays, particularly on later cognition.^1^ Expert consensus recommendations on neonatal nutrition intake exist^9^ but can be challenging to achieve in clinical practice, particularly in the sickest babies whose enteral intake may be constrained by a variety of disease processes.

Macronutrient analysis of human milk, both mothers’ own and donor, has been well described.^7,8,10,11^ This literature consistently highlights the nutrition variability of breast milk, and many authors have advocated individualized milk fortification to improve preterm nutrition management.^10,11^ For certain neonates, such individualized fortification offers potential benefits; however, for a significant minority, especially those with gastrointestinal compromise, an approach that avoids or minimizes any further manipulation of their milk, particularly with a bovine-based fortifier, is appealing.^4^

We have previously described the theoretical effect on energy intake of feeding high energy as opposed to low energy donor breast milk and suggested that targeted milk use might provide an alternative to, or avoid the need for, multicomponent fortification.^6^ In this article, we have developed this hypothesis and produced a pragmatic methodology for improving cumulative energy intake obtained from DEBM by categorizing and distributing milk according to energy content. We have demonstrated that the potential benefit from this targeted system increases with increased duration and volume of DEBM administration. Ideally, we would like to offer all babies energy-dense DEBM; however, the finite nature of our resource necessitates distribution according to intake volumes. Premature and/or sick neonates often receive small volume, “trophic” enteral feeds for reasons other than nutrition, and it was this cohort, who received concomitant parenteral nutrition, who we felt would be least disadvantaged by our approach.

Although we have demonstrated improvements in cumulative energy intake obtained from DEBM, we acknowledge that this represents only 1 aspect of nutrition. It is widely recognized that the protein content of all breast milk, mothers’ own as well as donor, is often insufficient to meet the requirements of growing preterm infants without fortification.^1^ Our previous work identified that even after fortification, only 61% of donated milk samples would meet recommended protein intake requirements, raising the possibility of individualized protein supplementation.^7^ To inform the development of our categorization system, we explored both the energy and protein content of our milk. We concluded that the relatively small variance in protein made it unsuitable for use as the defining categorization nutrient, particularly when compared to the variance seen in energy content.^7^

## Conclusion

It is clear that what we have postulated is based on retrospective analyses; however, these data offer proof of concept that improved energy intake by targeted milk distribution is achievable. What remains to be demonstrated is whether this system can be effectively introduced into routine practice and, in particular, whether it would have any effect on short-term and longer term measurable nutrition and clinical outcomes. The only way to answer these questions would be to test the hypothesis within the context of a randomized controlled trial.

## References

[1] Embleton ND Early nutrition and later outcomes in preterm infants. World Rev Nutr Diet. 2013;106:26-32. 23428677 10.1159/000342553

[2] Singhal A Cole TJ Fewtrell M Is slower early growth beneficial for long term cardiovascular health? Circulation. 2004;109(9):1108-1113. 14993136 10.1161/01.CIR.0000118500.23649.DF

[3] Schanler RJ Lau C Hurst NH Smith EO Randomized trial of donor human milk versus preterm formula as a substitute for mothers’ own milk in the feeding of extremely premature infants. Pediatrics. 2005;116(2):400-406. 16061595 10.1542/peds.2004-1974

[4] Cristofalo EA Schanler RJ Blanco CL Randomized trial of exclusive human milk versus preterm formula diets in extremely premature infants. J Pediatr. 2013;163(6):1592-1595. 23968744 10.1016/j.jpeds.2013.07.011

[5] American Academy of Pediatrics. Breastfeeding and the use of human milk. Pediatrics. 2012;129:e827-e841. 22371471 10.1542/peds.2011-3552

[6] Arslanoglu S Corpeleijn W Moro G Donor human milk for preterm infants: current evidence and research directions. J Pediatr Gastroenterol Nutr. 2013;57(4):535-542. 24084373 10.1097/MPG.0b013e3182a3af0a

[7] Cooper AR Barnett D Gentles E Macronutrient content of donor human breast milk. Arch Dis Child Fetal Neonatal Ed. 2013;98(6):F539-F541. 23867707 10.1136/archdischild-2013-304422

[8] Wojcik KY Rechtman DJ Lee ML Macronutrient analysis of a nationwide sample of donor breast milk. J Am Diet Assoc. 2009;109(1):137-140. 19103335 10.1016/j.jada.2008.10.008

[9] Agnostini C Buonocore G Carnielli V Enteral nutrient supply for preterm infants: commentary from the European Society for Paediatric Gastroenterology, Hepatology and Nutrition. J Pediatr Gastroenterol Nutr. 2010;50(1):1-9. 19881390 10.1097/MPG.0b013e3181adaee0

[10] De Halleux V Rigo J Variability in human milk composition: benefit of individualized fortification in VLBW infants. Am J Clin Nutr. 2013;98(2):529S-535S. 23824725 10.3945/ajcn.112.042689

[11] Aiko M Katsumi M Masahiko M Bedside analysis of human milk for adjustable nutrition strategy. Acta Paediatr. 2009;98(2):380-384. 19143668 10.1111/j.1651-2227.2008.01042.x

